# The synergistic antibacterial activity of ozone and surfactant mists

**DOI:** 10.1039/d3ra03346e

**Published:** 2023-07-26

**Authors:** Emmanuel I. Epelle, Neli Cojuhari, Abdalla Mohamedsalih, Andrew Macfarlane, Michael Cusack, Anthony Burns, Charles McGinness, Mohammed Yaseen

**Affiliations:** a School of Computing, Engineering & Physical Sciences, University of the West of Scotland Paisley PA1 2BE UK mohammed.yaseen@uws.ac.uk; b School of Engineering, Institute for Materials and Processes, The University of Edinburgh Sanderson Building, Robert Stevenson Road Edinburgh EH9 3FB UK; c ACS Clothing 6 Dovecote Road Central Point Logistics Park ML1 4GP UK

## Abstract

The microbiological safety of medical equipment and general surfaces is paramount to both the well-being of patients and the public. The application of ozone (a potent oxidant) has been recognised and implemented for this purpose, globally. However, it has primarily been utilised in the gaseous and aqueous forms. In this study, we investigate the potency of fine ozone mists and evaluate the synergistic effect when combined with cationic, anionic and non-ionic surfactants (dodecyl trimethyl ammonium bromide – DTAB, sodium dodecyl sulfate – SDS, alkyl polyglycoside – APG) as well as polyethylene glycol (PEG). Ozone mist is generated *via* a nebuliser (equipped with a compressed gas stream) and the piezoelectric method; whereas fabric substrates contaminated with *Escherichia coli* and *Staphylococcus aureus* are utilised in this study. Contamination levels on the fabric swatches are evaluated using agar dipslides. Compared to gaseous ozonation and aqueous ozonation (*via* nanobubble generation), the produced ozone mists showed significantly inferior antimicrobial properties for the tested conditions (6 ppm, 5–15 min). However, the hybrid mist-based application of ‘ozone + surfactants’ and ‘ozone + PEG’ showed considerable improvements compared to their independent applications (ozone mist only and surfactant mist only). The ‘ozone + DTAB’ mist had the highest activity, with better results observed with the micron-mist nebuliser than the piezoelectric transducer. We propose a likely mechanism for this synergistic performance (micellar encapsulation) and demonstrate the necessity for continued developments of novel decontamination technologies.

## Abstract 2

Micellar enclosures of ozone nanobubbles create a synergistic-antibacterial effect, more potent than the independent application of ozone or surfactant mists.

## Graphical abstract

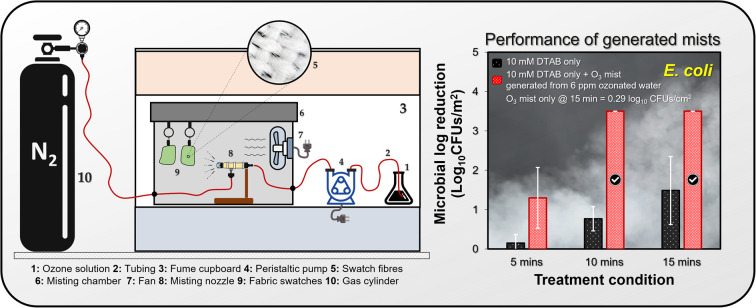

## 1. Introduction

The mitigation of microbial contamination on surfaces, equipment and medical devices is a crucial source of concern in several environments, particularly in hospitals where the risks of healthcare-associated infections (HAIs) are high. Intensive care units (ICUs) are the usual origins of these outbreaks since antibiotics administration here can be up to 10 times more than in general wards – resulting in the development of drug-resistant pathogens.^1,2^ The reduced effectiveness and the toxic by-products of conventional disinfectants have inspired the development of novel decontamination technologies, with a reduced probability of facilitating antimicrobial resistance, such as those involving ozone (O_3_). This interest has continued to grow over the past decade, as ozone typically decomposes to oxygen, and its interaction with organic compounds usually results in non-toxic by-products.^3^ The mechanisms of ozone's action are well documented and can be generally classified into direct oxidation (*via* ozone's interaction with the cell's components) and indirect oxidation involving bacterial exposure to reactive oxidative species (ROS).^4,5^ The resultant effect of both routes includes the rupturing of the cell membrane and eventual cell lysis, protein oxidation, DNA damage and the disruption of enzymatic activities. However, the bactericidal effects of these short-lived ROS are mainly evident when an imbalance exists between ROS exposure and the bacteria's antioxidant defences (oxidative stress).^6,7^ Several types of bacteria possess enzymes, such as catalases, peroxidases, and superoxide dismutases, which enable them to counteract the harmful impact of ROS.^8^ Higher-level exposures to ROS can disturb the homeostatic balance of the applied oxidant dosage and the rate of antioxidant production by the bacteria; thus, overpowering their defence mechanisms and resulting in their death. The consequent morphological changes to the cells include protrusions (resembling blisters), invaginations, leaked cell contents and the development of cell debris after rupture; the following studies^9–12^ provide extensive visualizations of these effects on different microorganisms.

**Fig. 1:**
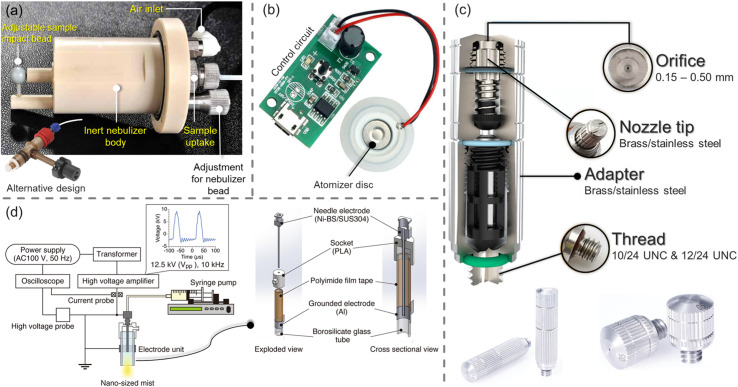
Methods of mist generation *via* (a) micron-mist nebulisers utilising a compressed gas stream (*e.g.* air or N_2_);^30,31^ (b) piezoelectric transducers that vibrate at high frequencies (up to 2.4 MHz) when an electric current is applied to them^32^ (c) high-pressure misting nozzles requiring liquid delivery pressures between 40 and 70 bar, with flow rates depending on the diameter of the nozzle^33^ and (d) dielectric barrier discharge (a recently proposed technique for generating nano-sized mists).^34^

The efficacy of ozone decontamination depends on several additional factors, which have been categorized into intrinsic and extrinsic factors.^12^ While intrinsic factors relate to the growth stage, cell envelope, and the efficiency of the microorganism's repair mechanisms, extrinsic factors include the ozone concentration and the presence of ozone-consuming organic materials in the decontamination environment. Another classification by Epelle *et al.*^10,13^ featured 3 main groups of influencing factors – ambient conditions, the nature of contaminated material/substrate and operational factors. The method of ozone application (gaseous, aqueous, or in the form of mists and droplets) is a crucial operational factor that is, in turn, dependent on cleaning and drying requirements and the desired penetration efficiency, particularly when objects of narrow/complex geometries are to be treated.^14^ An extensive discussion of the necessary considerations for the adoption of gaseous or aqueous ozonation is presented elsewhere.^15^ These considerations are based on a detailed analysis of several reports on the efficacy of ozone applied to different material surfaces in gaseous and aqueous forms.^16,17^ Non-wetting or slightly wetting ozone mists and sprays, generated from ozonated water, have been hardly utilized compared to gaseous and aqueous applications. The necessity to maintain milder treatment conditions (than with gaseous ozonation), increase material compatibility and eliminate the need for long drying regimes (as with immersion in aqueous ozone) have led to recent developments in moderately wetting spray devices and non-wetting fine mist generation technologies (Fig. 1) for disinfection applications. Some new technologies have also featured the use of electrostatic sprays that attract the aerosols to surfaces *via* electrostatic forces (75 times greater than gravitational forces).^18,19^ Thus, it is possible to tailor the generated mist particles to target the desired contaminants, depending on the nature of the surface. Furthermore, aqueous and mist-based ozone applications have the added advantage of reduced human toxicity compared to gaseous ozonation. Most ozone therapies (*e.g.* in dentistry) employ ozonated water due to its antimicrobial potential *in vitro* and *in vivo*.^20,21^ The use of ozone droplets and mists has been successfully applied at 2 and 4 mg L^−1^ for the inactivation of phytopathogenic bacteria (*Agrobacterium tumefaciens*, *Erwinia amylovora* and *Pseudomonas syringae*).^22^ Oliveira *et al.*^23^ evaluated the efficacy of an ozonated water spray chamber for the decontamination of personal protective equipment infected with *Gammacoronavirus*. Aqueous ozone concentrations between 0.3 and 0.9 mg L^−1^ were adopted, with 100% microbial reduction obtained in some tested cases. The authors extended their work to capture the perception of the public when the spray chamber was used to decontaminate garments and accessories actively worn by participants/volunteers; up to 2.4 log reduction was achieved, although with wetting observed.^24^

Despite these advancements, a key challenge of oxidation-based techniques, as with many other disinfection methods is the potential for incomplete microbial inactivation, and this makes the combined usage with other disinfectants recommendable.^7,25^ The synergistic effect of ozone when combined with other chemicals/disinfectants has been investigated by several researchers. de Souza and Daniel^26^ have demonstrated the enhanced inactivation of *E. coli* when a combined but sequential treatment involving ozone and chlorine is administered for water disinfection. This observation was applicable to all applied dosages in their work. Additionally, the combination of peracetic acid (PAA – another widely-applied disinfectant) and aqueous ozone has been found to be effective in killing *Campylobacter jejuni*, *Salmonella typhimurium*, and *E. coli* bacteria. It was realised that the combination of aqueous ozone (10 ppm) also reduces the amount of vaporized PAA (500 ppm) in the surrounding environment and thus minimizes the health risks associated with handling PAA.^27^ More recently, Britton *et al.*^28^ showed that the antimicrobial efficacy of aqueous ozone can be increased when combined with short-chain fatty acid buffers compared to their independent applications. The creation of an acidic environment using buffer systems reduced the survival rate of *S. aureus* (through the degradation of the cell's cytoplasm) and increased ozone stability for sustained antimicrobial action. Epelle *et al.*^29^ successfully demonstrated the complementary effect of gaseous ozone and UVC for the decontamination of a wide range of microbes (*E. coli*, *S. aureus*, *Candida albicans* and *Aspergillus fumigatus*) on several materials (stainless steel, textiles, copper, PMMA, and facemasks).

In addition to ozone, surfactants which typically eliminate soil from surfaces (cleaning) are also recommended for disinfection in healthcare environments as a result of their antimicrobial properties.^35^ The hydrophobic (water-repelling) portion of surfactant molecules^36^ can penetrate the cell membrane of bacteria and interact with the lipids therin, causing the membrane to become disrupted. This in turn leads to increased permeability and ultimately, the death of the cell. As with ozone, mist-based applications of surfactants are scarce as they are mainly applied in the aqueous phase for cleaning purposes. In this study, the antimicrobial efficacy of ozone and surfactant mists (cationic, anionic and non-ionic) are independently explored and the potential for their synergistic action on Gram-positive and Gram-negative bacteria is investigated for the first time. The initial hypothesis of this study was that the application of ozone-stabilizing properties of surfactants as documented in our previous investigation^37^ coupled with the combined antimicrobial properties (of ozone and the surfactant) will facilitate the degradation of the tested bacteria; thus making for a more rapid treatment process for surfaces and medical devices. As is subsequently shown, this synergistic application has the potential to alter the surface charge of the mist, thereby improving the inactivation efficacy of the target organisms.

## 2. Methodology

### 2.1 Substrate preparation

**Fig. 2:**
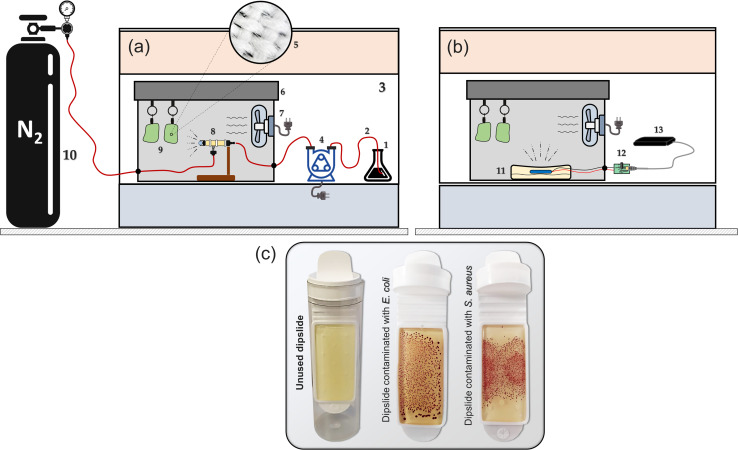
Experimental arrangements for the (a) compressed gas method (micron-mist nebuliser – Fig. 1(a) and (b) the piezoelectric method (Fig. 1b). The annotated components include: 1: ozone solution, 2: tubing, 3: fume cupboard, 4: peristaltic pump, 5: swatch fibres, 6: misting chamber, 7: mist circulation fan, 8: mist generator, 9: fabric swatches, 10: gas cylinder, 11: dish containing the desired mixture and the piezoelectric transducer, 12: control circuit, 13: power supply. (c) Shows both unused and contaminated dipslides.

A representative colony of the bacteria (*E. coli* NCTC 12900 or *S. aureus* ATCC 25923) was added to 10 mL of nutrient broth (Sigma-Aldrich in St. Louis, USA). The mixture was then incubated in a shaker at 37 °C and 150 rpm for 14 hours. Subsequently, 1 mL of the suspension was centrifuged in a microcentrifuge tube at 10 000 rpm for 7 minutes. The harvested cells were washed with a 0.01 M phosphate buffer saline (PBS) solution, and the suspension's absorbance was measured at 570 nm. The absorbance was adjusted to an optical density (OD) of ∼0.01, which corresponds to 10^8^ cells per mL for *E. coli* and 10^7^ cells per mL for *S. aureus* bacteria. Sterile fabric swatches (35% cotton and 65% polyester) were inoculated with 100 μL of the respective bacterial suspensions prepared and then subjected to mist treatments. Agar dipslides^38^ were applied to the swatches and incubated at 37 °C for 24–48 hours to evaluate growth levels before and after treatment (Fig. 2c). MATLAB (R2020b) was used to post-process images of the agar dipslides and determine the contaminated area fraction as well as the number of formed colony units by the bacteria. To establish the control (before treatments), the number of recoverable viable colony-forming units (CFUs) per cm^2^ of the fabric swatches was enumerated from the dipslides according to the procedures described in our recent study.^29^ Approximately 3500 CFUs per cm^2^ were counted (*via* computer program written in MATLAB) on the *E. coli* slide whereas, up to 5800 CFUs per cm^2^ of *S. aureus* were enumerated from the control experiments; similar enumeration techniques have been used in the following study.^39^

### 2.2 Mist treatment

To generate ozone mists from ozonated water, mineral water^37^ was initially ozonated *via* an electrolysis oxygen radical generator (EORG™ – Novus Clean Tech Ltd, UK) until a concentration of 6 ppm was attained as determined by the Palin test procedure;^40^ the EORG device mainly generates ozone nano/microbubbles in the size range of ∼100–1000 nm from Dynamic Light Scattering (DLS) measurements as previously documented in our study.^5^ The generated ozonated water was immediately utilised for mist production according to the methods shown in Fig. 1a, b and 2a, b; however, the amount of ozone in the generated mist was not measured, as a result of humidity constraints of our gaseous ozone monitor. The compressed gas method^41^ (Fig. 1a and 2a), involved the passage of nitrogen gas at 2 bar, to a nebulizer simultaneously receiving the ozonated solution at a flow rate of 350 mL h^−1^. While higher pressures could be utilised to generate finer mists, careful attention must be paid to the pressure limits of the utilised tubing to avoid rupture or disentanglement.

**Fig. 3:**
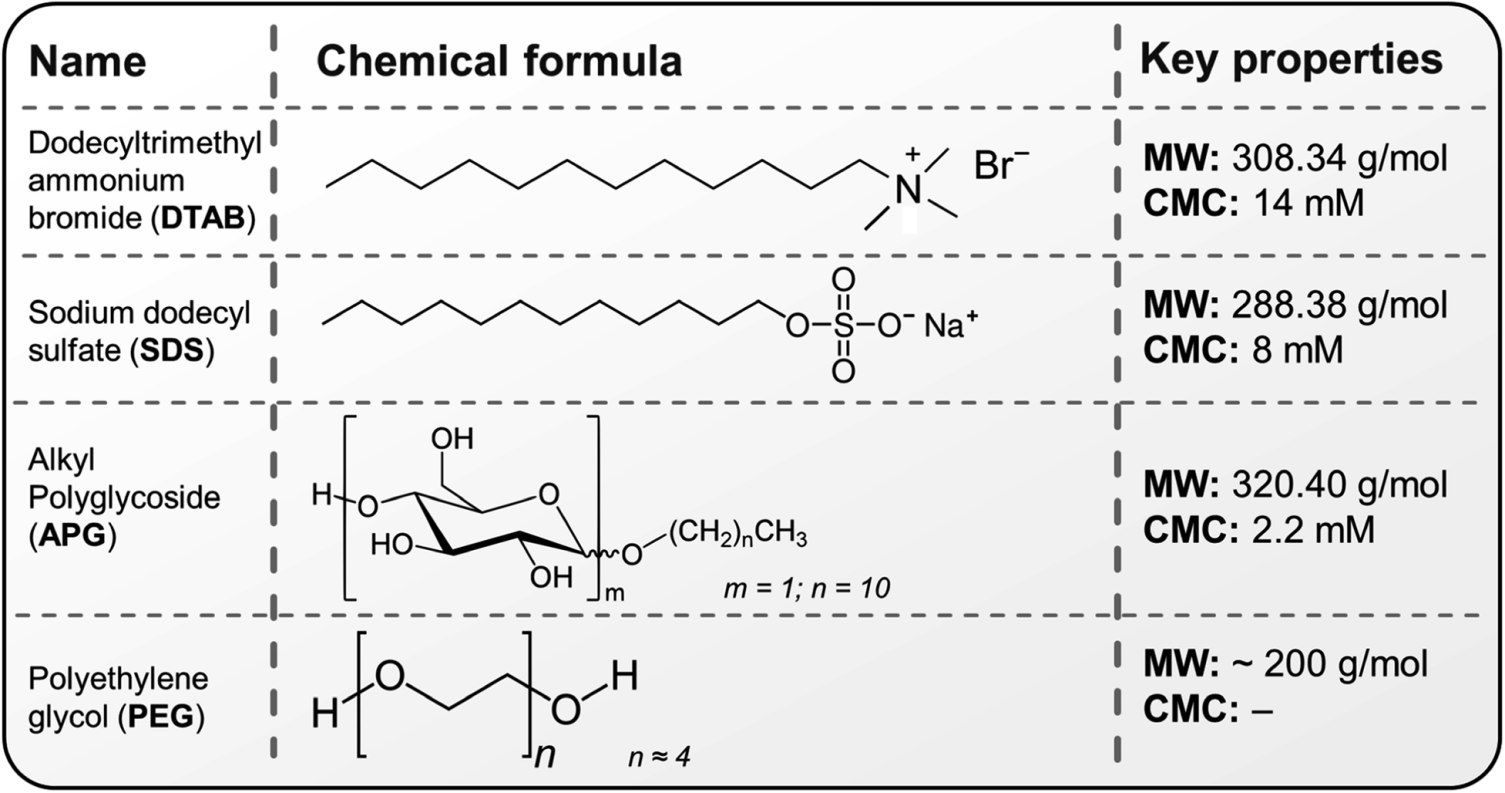
Chemical structure and properties of the surfactants and polymers utilised in this study; CMC represents the critical micelle concentration, which is the concentration above which surfactant micelles begin to form in solution. The CMCs of the above surfactants in water are obtained from the following sources.^43,44^

With the piezoelectric method (Fig. 2b),^42^ the transducer/atomiser disc is placed in the solution (contained in a dish), which is situated in the misting chamber. The arrangement (Fig. 2b) enables the device to be controlled externally, *via* the switch on the circuit (Fig. 2b). When it was desired to create a mist of ‘ozone + surfactants’ and ‘ozone + polymer’, 10 mM solution (the desired concentration) was achieved by transferring the required mass/volume of the chemicals into 150 mL of ozonated water at 6 ppm (this volume was sufficient for continuous mist generation over the longest duration employed in this study – 15 min). It was crucial that the chosen surfactant and polymer possessed an almost instantaneous solubility in water, as this eliminated potential downtime, that would have facilitated ozone decomposition. Fig. 3 provides the chemical structure, molecular weight and critical micelle concentrations (CMC) for the different surfactants. It can be observed that the 10 mM concentration utilised throughout this study is above the CMC of all surfactants except DTAB. The effectiveness of the mist treatment was primarily evaluated *via* log reductions in the viable CFU counts recorded on the dipslides and can be estimated according to eqn (1). All tests were conducted in triplicates and the standard deviation, reported as error bars.1
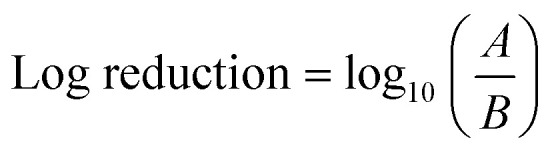
where *A* is the number of CFUs before treatment and *B* is the number of CFUs after treatment.

## 3. Results and discussion

Compared to the non-ozonated solutions, the ozonated solutions had a lower pH – indicating the accompanied production of acidic by-products such as organic acids and hydrogen peroxide, particularly as the generated ozone decomposes (Table 1). It can also be observed that the addition of surfactants to the ozone solution slightly increases the pH compared to pure ozonated water. It has been reported that the presence of OH^−^ ions (increased alkalinity), facilitates ozone decomposition in aqueous solutions.^45^ This process as described by the reaction mechanisms of Tomiyasu *et al.*^46^ produces further ROS, which are short-lived in solution, although with significant degradative impacts on bacteria. Moreso, the improvement of ozone stability by surfactants in solution has been previously observed.^37,47^ Thus, it can be argued that surfactants play a dual role, with regard to ozone stability; both of which contribute to microbial inactivation efficacy as described subsequently; this dual role is also a function of the surfactant concentration. It is also worth mentioning that the method of aqueous ozone generation utilised herein involves the production of ozone nanobubbles *via* electrolysis, and this also contributes to the ozone stability compared to the generation of predominantly micro-sized bubbles. Ozone nanobubbles generally have a longer residence time in aqueous solutions compared to larger macrobubbles, which tend to quickly rise to the air–water interface and then collapse. This extended duration of ozone nanobubbles in aqueous solutions can be attributed to their greater gas–liquid interfacial area, electrostatic repulsion (due to their net negative charge – zeta potentials as reported in Epelle *et al.*^5^), decreased buoyancy, Brownian motion and resistance to coalescence.^48,49^

**Table 1:** pH of tested solutions utilised for mist generation^a^

Solution	pH
*Mineral water only*	*7.98*
10 mM DTAB solution	8.22
10 mM SDS solution	8.26
10 mM APG solution	10.05
10 mM PEG solution	8.09
*6 ppm ozonated water only*	*7.32*
(6 ppm ozonated water + 10 mM DTAB) solution	7.42
(6 ppm ozonated water + 10 mM SDS) solution	7.36
(6 ppm ozonated water + 10 mM APG) solution	9.87
(6 ppm ozonated water + 10 mM PEG) solution	7.47

a All solutions were prepared using mineral water with ionic composition provided in ref. 37. This mineral water was also utilised for ozone generation.

### 3.1 The efficacy of ozone mists

**Fig. 4:**
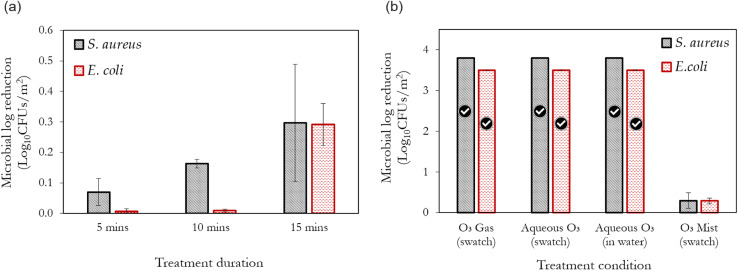
(a) The antimicrobial efficacy of ozone mists generated from (6 ppm ozonated water) over a treatment duration of 5, 10 and 15 min using the compressed gas method; (b) a comparison of the antimicrobial efficacies of ozone mists, gaseous ozonation (6 ppm) and aqueous ozonation (6 ppm), after 15 min of treatment. The tick marks represent 100% bacteria removal which correspond to >3.8 log_10_ reduction for *S. aureus* and >3.5 log_10_ reduction for *E. coli*, respectively. *Aqueous O*_*3*_*(swatch)* represents the microbial reduction on the swatch after treatment whereas, *Aqueous O*_*3*_*(water)* represents the microbial reduction in the water used for treatment.

Fig. 4a illustrates the efficacy of ozone mists generated *via* the compressed gas method (Fig. 2a) over time. Despite the increasing treatment efficiency, it can be observed that less than 1 log_10_ reduction is achieved, even at the longest treatment duration utilised. This poor performance may be attributed to hydrodynamic cavitation (the formation, growth and collapse of microbubbles) as a result of the rapid change in pressure upstream and downstream, the nebuliser.^50^ While it may be argued that the pressure changes utilised in compressed gas nebulisers (where the liquid is atomised by the energy of the high-velocity gas stream) may not be sufficient to cause cavitation, it is worth noting that this pressure change across the nebuliser can cause the dissolved ozone gas to come out of solution and form bubbles. This effect is also complemented by ozone's low solubility in water. Thus, pressure fluctuations induce a disruption of the gas–liquid equilibrium and can cause bubble formation and collapse in the nebuliser. While this process is crucial for the formation of mists, it is in turn detrimental to the stability of ozone. This phenomenon also occurs with high-frequency sound waves (acoustic cavitation/ultrasonication) and thus can be regarded as the plausible explanation for the reduced efficiencies also observed with the piezoelectric mist generation method (shown subsequently). Jyoti and Pandit also demonstrated the detrimental effect of cavitation on aqueous ozone stability.^51^ These observations constitute possible reasons why ozone mists are less prevalent compared to vapourised hydrogen peroxide (H_2_O_2_) solutions, as H_2_O_2_ has a significantly higher solubility in water than ozone. Hence, the development and application of ozone stabilisation methods are crucial if the full benefits of ozone are to be realised during mist-based applications at low concentrations.

Conversely, it can be observed that gaseous and aqueous ozonation of the contaminated swatch yielded 100% bacterial removal at the same conditions utilised for the misting process (6 ppm and 15 min) – Fig. 4b. To determine the potential transfer and survival of bacteria from the contaminated swatch to the water in which the swatch was immersed during aqueous ozonation, the dipslides were used to test the water (*Aqueous O*_*3*_*in water* – Fig. 4b). No viable bacterial growth was observed. Interestingly a similar observation was reported by Tanuwidjaja and Fuka *et al.*^22^ They realised that the use of ozone droplets (*via* a low-pressure sprayer) was more efficient for the inactivation of plant pathogens than ozone's application in the mist form. The low adherence and penetration of the generated mists to the contaminated surfaces is another potential contributing factor to the poor performance of ozonated mists. Although mist sprayers may produce better coverage of the disinfected area compared to heavier wetting droplets, it has been demonstrated that mist sprayers can limit the adherence of the generated mists to the contaminated surfaces.^52^ Based on this observation, the inferior performance of the misting process (reported here) in comparison to gaseous ozonation (with excellent penetration properties), and full immersion during aqueous ozonation, can be better understood. However, Cabral *et al.*^53^ recently demonstrated the effectiveness of ozone mists for the inactivation of *E. coli*. Although the exact method of mist generation was not mentioned, the high ozone concentrations (up to 51 ppm) utilised in their study is the key difference compared to this study (6 ppm). Thus, higher dissolved ozone concentrations than that utilised in this study are required to achieve significant antimicrobial activity from ozone mists. It should be pointed out that large-scale applications of aqueous ozone hardly realise concentrations >10 ppm due to mass transfer limitations. Furthermore, the use of ozone gas in highly humidified environments, although different to direct misting from ozonated water, has been shown to have good decontamination potential.^54^ Such endeavours require close monitoring of ozone stability, as ozone tends to decompose more rapidly under humid conditions.^15^

It is also important to highlight the vital role that the initial microbial load plays in the inactivation by ozone mists. As part of our preliminary experiments, an initial bacterial suspension with an OD of 0.2 was utilised (corresponding to 10^9^ cells per mL) for inoculating the fabric swatches. No identifiable improvements were observed when ozone mists generated by either method was applied; however, gaseous and aqueous ozonation at the same conditions (6 ppm, 15 min) yielded 100% removal. This dependence on the initial bacterial load has also been demonstrated in the following studies.^22,55^ This establishes the importance of immediate and frequent disinfection or sterilisation, to prevent the build-up of bacteria to levels that may be too difficult to satisfactorily eliminate.

### 3.2 The synergistic effect

**Fig. 5:**
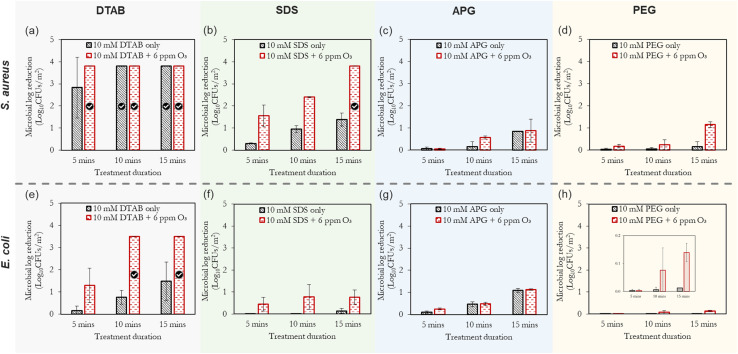
The combined effect of ‘ozone + surfactants’ mists and ‘ozone + PEG’ mists on the inactivation efficiency of *S. aureus* (a–d) and *E. coli* (e–h). The compressed gas method is used here. The tick marks represent 100% bacteria removal which correspond to >3.8 log_10_ reduction for *S. aureus* and >3.5 log_10_ reduction for *E. coli*, respectively. As shown in Fig. 4, the application of only ozone mists at the longest duration (15 min) yielded only 0.3 log_10_ reduction for *S. aureus* and 0.29 log_10_ reduction for *E. coli*.

It can be observed in Fig. 5 that the combination of ‘ozone + surfactants’ and ‘ozone + polymer’ generally outperforms the inactivation efficacy of the surfactant alone. This inactivation effect increases with time, with *S. aureus* (Gram-positive bacteria) showing a marked sensitivity to the treatments compared to *E. coli* (Gram-negative bacteria). This observation (increased sensitivity of *S. aureus* over *E. coli*) is corroborated by other researchers.^56,57^ The ‘ozone + DTAB’ mist is the best-performing combination, and this was followed by combinations involving SDS, APG and PEG, respectively. The positively-charged ammonium groups of cationic surfactants interact with the negatively-charged cell membrane of the bacteria, thereby disrupting its structure and causing cytoplast leakage.^57^ Additionally, DTAB is capable of forming self-assembles below the CMC, which can solubilise lipids of the microbial cell membrane.^58^

Anionic surfactants, which have a negatively charged hydrophilic head can electrostatically interact with the lipid components of the cell membrane as well as the positively charged amino acid residues in proteins, causing their denaturation.^35^ Non-ionic surfactants (*via* their hydrophobic chains) can interfere with the hydrophobic components of the cell membrane and its internal cellular enzymatic activity; thus, limiting the proliferation of the bacteria. Furthermore, compared to surfactant concentrations below the CMC, the formation of micelles enhances the antimicrobial potential of surfactants by increasing the local concentration of the hydrophobic tails, improving contact with microorganisms, providing stability, and enhancing solubilization and delivery of hydrophobic agents to target contaminants. However, as will be subsequently discussed, the significance of these effects tends to be dependent on the type of bacteria.

Compounds with alkyl groups (*e.g.*, surfactants), at low concentrations, typically act as OH˙ radical scavengers; thus, reducing the rate of ozone decomposition – this is the likely mechanism governing the increased ozone stability and consequent antimicrobial activity in scenarios where the surfactant concentration was below the CMC (the case of DTAB). Whereas, at higher concentrations (above the CMC, as in the case of SDS and APG), the observed ozone stabilisation and activity can be attributed to both the OH˙ scavenging effect and the micellar/vesicular enclosure of ozone. The rationale for this proposition of micellar enclosure is that ozone is a hydrophobic molecule and is more likely to dissolve in hydrophobic solutions. Eriksson *et al.*^47^ highlighted that sugar-based molecules (*e.g.* starch and dextran) which are similar to the structure of APG can create a complex with aqueous solutions that can encapsulate ozone, making it more stable and soluble. This complex is thought to form due to increased electron density on the central oxygen atom, which creates a resonance structure with a δ^+^-terminal oxygen atom and a δ^−^-terminal oxygen atom. The overall consequence of both scenarios (OH˙ radical scavenging and enclosure/encapsulation) is the increase in the direct oxidation of the microbial cells (by ozone) over indirect oxidation. The described encapsulation mechanism bears considerable resemblance to vesicle drug delivery,^59,60^ where ozone is stabilised and transported to the target sites of microbial contamination.

**Fig. 6:**
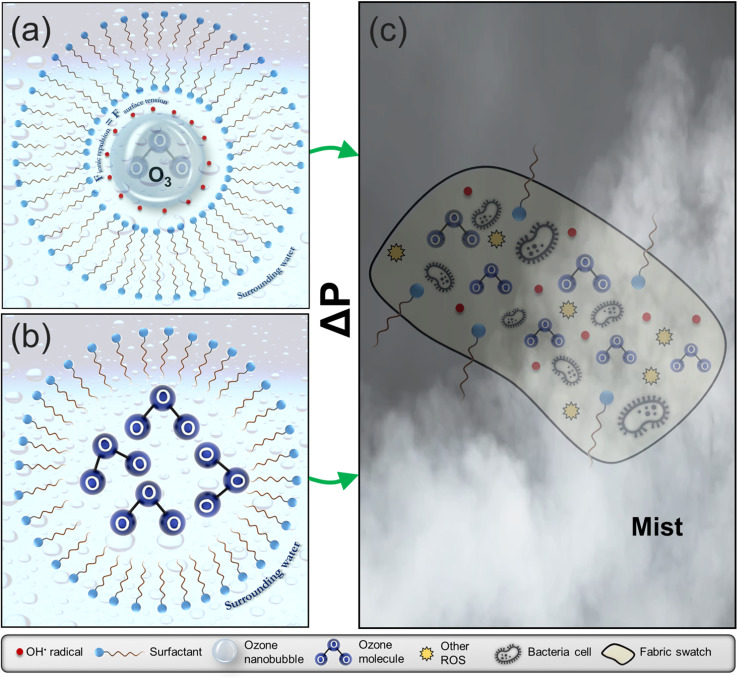
A representation of the encapsulation of ozone nanobubbles by (a) surfactant bi-layer vesicles (b) surfactant micelles in water, and the (c) corresponding inactivation by free radicals, ozone, and alkyl hydrophobic surfactant chains.

Additionally, the surface tension reduction properties of surfactants also help to spread droplets of a mist more evenly over a surface, reducing the likelihood of the droplets coalescing or running off the surface. This increased adherence can be useful in applications such as cleaning, where it is important to ensure that the mist reaches all areas of the contaminated surface. Another complexity that arises in the case of ozone encapsulation is the interaction mechanism between the generated ozone nanobubbles and surfactant micelles. Fig. 6. The formation of vesicular enclosure (Fig. 6a) is a probable mechanism of ozone stabilisation as the hydrophilic surfactant head orients towards the aqueous environments (the surrounding water and the nanobubble). For micellar enclosure (Fig. 6b), the ozone molecules can be directly protected by the micelles formed in the solution. Without these protective mechanisms, the resultant observation is a degassing effect (removal of the ozone from water and its rapid decomposition to oxygen atoms as a result of the high pressure drop and ultrasound effects experienced by the molecules during mist production).^61,62^ According to Fig. 5a and e, the minimum inhibitory treatment duration for the combined (10 mM DTAB + 6 ppm ozone scenario) is less than 5 min for *S. aureus*; whereas for *E. coli*, it is between 5 min and 10 min. This illustrates the rapid inactivation kinetics of this mixture in the form of mists.

**Fig. 7:**
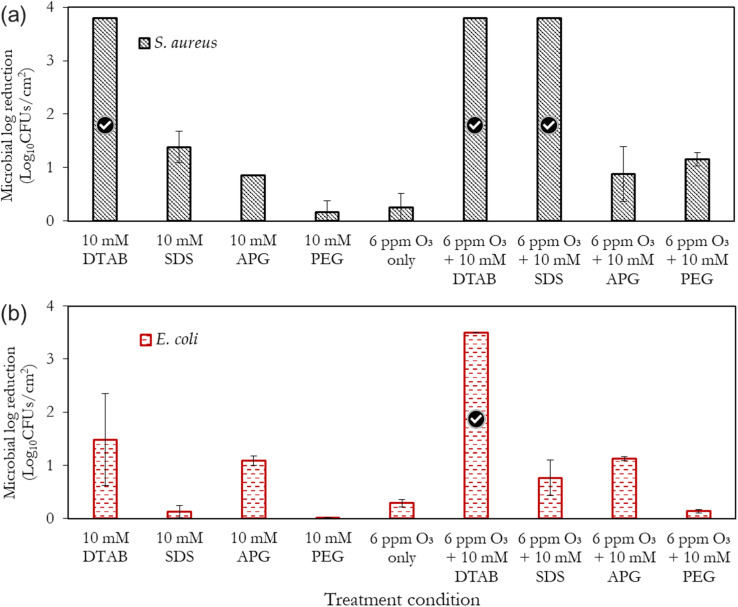
Performance summary of the different formulations utilised for the mist-based inactivation of (a) *S. aureus* and (b) *E. coli*. The treatment duration is 15 min using the compressed gas method. The tick marks represent 100% bacteria removal which corresponds to >3.8 log_10_ reduction for *S. aureus* and >3.5 log_10_ reduction for *E. coli*, respectively.

Fig. 7a and b present an overall summary of the independent and combined treatments for both bacteria. With *S. aureus*, the sole application of the surfactants and polymer was in the order DTAB > SDS > APG > PEG. This order follows the relative performance of cationic, anionic and non-ionic surfactants as documented in ref. 35. With *E. coli*, however, the observed trend is different, with the APG mists showing a marked ability to inactivate this bacteria compared to SDS. The outer membrane of Gram-negative bacteria is composed of lipopolysaccharides that contain hydrophilic and hydrophobic domains, with an overall negatively-charged cell membrane^57^ compared to Gram-positive bacteria. APG, which has a non-ionic hydrophilic head and a hydrophobic tail, may have better penetration and interaction with the cell wall of *E. coli* than SDS, which is also negatively charged. On the other hand, *S. aureus* has a thicker peptidoglycan layer in its cell wall, which may make it less permeable to the larger-sized APG molecules. Therefore, SDS, which has a smaller molecular size, may be more effective in disrupting the cell wall of *S. aureus*.

Remarkably, PEG showed considerable ozone-stabilizing characteristics during *S. aureus* treatment (possibly *via* the formation of complexes and micelles) (Fig. 7a). The oxygen groups in the PEG polymer have the potential to form hydrogen bonding between the lone pairs of the ozone molecule and the polymer network, thus facilitating ozone encapsulation. However, its combined performance with ozone for the decontamination of *E. coli* was low (albeit better than PEG alone). Gram-negative bacteria have a thin peptidoglycan layer surrounded by an outer membrane containing lipopolysaccharides, while Gram-positive bacteria, have a thick peptidoglycan layer but lack an outer membrane. The presence of the outer membrane in Gram-negative bacteria may have made *E. coli*, generally more resistant to the mist treatments than Gram-positive bacteria, *S. aureus*. Although this observation is similar to the previous reports,^56,57^ further investigations are required to elucidate this peculiar difference. It can also be observed that the inactivation efficacy of DTAB on *S. aureus* was as good as its combination with ozone (Fig. 7a). A separate investigation will be pursued involving the use of different surfactant concentrations well above and below the CMC, to rigorously quantify the relative antimicrobial contributions of the independent treatments (surfactant only) compared to the combined synergistic treatments as well as the minimum inhibitory ozone concentrations. Nonetheless, this study represents the first step towards fully understanding this synergistic phenomenon.

### 3.3 A comparison of the piezoelectric and compressed gas methods

**Fig. 8:**
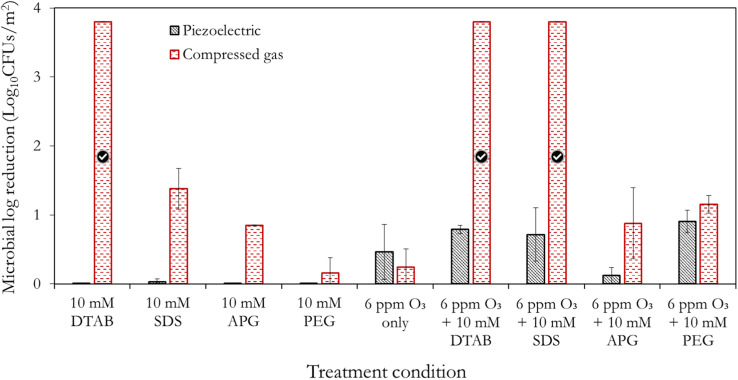
A comparative summary of the mist-based inactivation efficacies of the piezoelectric and the compressed gas methods after 15 min treatment; swatches were contaminated with *S. aureus*. The tick marks represent 100% bacteria removal which correspond to >3.8 log_10_ reduction for *S. aureus*.

As with the compressed gas method, the synergistic effect of the surfactants and polymer with ozone is demonstrated with the piezoelectric method, relative to their independent applications (Fig. 8) – thus substantiating the previous observations. It is also evident from Fig. 8, that the use of the compressed gas method dominates the antimicrobial activities obtained by the piezoelectric method. Importantly, no antimicrobial effects were observed with the surfactant-only/polymer-only scenarios. This is indicative of some separation occurring below the atomiser disc so that mainly water is aerosolised during the piezoelectric mist treatment. Interestingly, Dehghani *et al.*^63^ demonstrated that ultrasound can be utilised for the degradation of anionic surfactants in water, and thus supports our observation. It is thus hypothesized that there could be a threshold concentration for which the surfactants are effectively aerosolised by the piezoelectric method – a subject of further investigation, as previously indicated.

Furthermore, the piezoelectric mist generation method induces more significant local pressure differences which lead to higher cavitation effects and in turn higher degassing compared to the compressed gas method. This implies that the compressed gas method retains more ozone in the solution and thus yields better antimicrobial action. Additionally, the use of the compressed gas method for 15 min resulted in fabric swatches that were slightly wet compared to the piezoelectric method which produced finer mists. This implied better adherence/penetration of the produced droplets to the surface of the fabric swatches. Thus, the combination of the earlier highlighted separation effects beneath the transducer plate, increased cavitation and the difference in the droplet size, are the likely reasons for the enhanced performance of the compressed gas method. By comparing the combined piezoelectric treatments, it can be seen ‘ozone + PEG’ shows good potential relative to other chemicals. These results indicate that the method of mist generation is crucial to exploiting the potencies of antimicrobial agents, and should not be overlooked during the design of decontamination systems. While 2 of the mist generation methods highlighted in Fig. 1a and b, have been explored in this study, further work could involve the application of other generation methods including those in Fig. 1c and d to determine the existence of potential anomalies or improved inactivation efficacies. New methods that address the limitations of these mist generation methods are also needed to increase the versatility of existing decontamination equipment as well as facilitate the design of new ones.

## 4. Conclusion

It was realized that the application of ozone mists generated from 6 ppm ozonated water, resulted in a significant reduction of the antimicrobial efficacy compared to the direct application of aqueous ozone on the fabric swatches (*via* immersion). This poor performance of the ozone mists only, relative to full immersion in aqueous ozone or gaseous ozonation under the same conditions, was attributed to the degassing effect *via* rapid pressure changes and cavitation during the mist generation process. However, our study shows that it is possible to create effective cleaning formulations by combining surfactant compounds with ozone in the form of mists – with better inactivation characteristics compared to the independent applications of ozone and surfactant mists. Regardless of the misting approach adopted (nebulization *via* a compressed gas stream or a piezoelectric transducer), a synergistic effect between surfactant and ozone mists was observed; nevertheless, the compressed gas method proved more consistent and effective. We hypothesized that the surfactant helps to stabilize the ozone in the mist (*via* vesicular and micellar encapsulation), allowing for more efficient delivery of ozone to the target surface harbouring the microorganisms. We plan to advance our investigations by utilising robust imaging procedures (*e.g.* Cryo-TEM) for further verification of this encapsulation mechanism. Additionally, the surfactant helps to increase the contact time/adherence between the ozone mist and the microorganisms on the contaminated surface, leading to greater antimicrobial efficacy. Another possibile reason for the synergistic activity highlighted is the OH˙ radical scavenging properties of surfactants, which help to retard ozone decomposition. Additionally, the overall disinfection process benefits from the antimicrobial properties of the surfactant, themselves. The ‘ozone + DTAB’ mist possessed the highest activity compared to the other ozone mixtures containing (SDS, APG and PEG, respectively). The development of eco-friendly surfactants with antimicrobial properties, which can be synergistically applied in the form of mists is crucial for the sustainability of decontamination operations in several industries and should thus be further pursued. More developments are also required for accurate measurement of ozone mists generated from ozonated water (*i.e.* ozone sensors compatible with very high-humidity environments).

## Abbreviations

APG: Alkyl polyglycosides
CFU: Colony forming unit
DTAB: Dodecyl trimethyl ammonium bromide
DLS: Dynamic light scattering
EC: *Escherichia coli*
HAI: Healthcare-associated infections
ICU: Intensive care unit
OD: Optical density
PEG: Polyethylene glycol
ROS: Reactive oxidative species
SA: *Staphylococcus aureus*
SDS: Sodium dodecyl sulfate

## Author contributions

Emmanuel I. Epelle: conceptualization, methodology, investigation, software, data curation, writing original draft, writing review draft. Neli Cojuhari: methodology, investigation. Abdallah Mohamedsalih: methodology, data curation. Andrew Macfarlane: writing review draft, funding. Michael Cusack: writing review draft, funding. Anthony Burns: writing review draft, funding. Charles McGinness: methodology, investigation. Mohammed Yaseen: conceptualization, methodology, writing review draft, funding, lead & PI.

## Conflicts of interest

The authors declare that they have no known competing financial interests or personal relationships that could have appeared to influence the work reported in this paper.

## Supplementary Material
